# Applying the COM‐B Model to Identify Barriers to Bulevirtide Adherence in Chronic Hepatitis D: A Multicenter Italian Study

**DOI:** 10.1111/jvh.70097

**Published:** 2025-10-11

**Authors:** Lorenzo Badia, Alessia Ciancio, Marco Distefano, Antonio Izzi, Alessandro Loglio

**Affiliations:** ^1^ Infectious Diseases Outpatient Clinic Azienda USL of Imola Imola Italy; ^2^ Department of Medical Sciences Molinette Hospital Turin Italy; ^3^ UOSD Hepatology Unit ASP Siracusa Siracusa Italy; ^4^ Department of Infectious Diseases Cotugno Hospital Naples Italy; ^5^ Gastroenterology, Hepatology and Transplantation Division ASST Papa Giovanni XXIII Bergamo Italy

**Keywords:** behavioural science, bulevirtide, COM‐B model, HDV adherence, motivation, treatment barriers

## Abstract

Adherence to treatment is a key determinant of clinical outcomes in chronic infectious diseases, including hepatitis D virus (HDV) infection. Even if bulevirtide (BLV) has shown a promising adherence profile in clinical trials, adherence is often compromised by various barriers in the context of HDV—especially among migrant populations. The aim of this study was to investigate the factors influencing adherence to BLV treatment in real‐life settings in Italy. From May to September 2024, two anonymous surveys—one for HDV patients undergoing bulevirtide (BLV) treatment and one for their hepatologists—were conducted across five tertiary centers in Italy. The study employed the COM‐B model (Capability, Opportunity, Motivation–Behaviour) to systematically explore the behavioural drivers influencing treatment adherence in this population and unmet needs. Of the 86 consecutive adult patients receiving bulevirtide (BLV) who were invited to participate, 83 (97%) completed the multilingual survey (35% > 60 years old, 48% Italians; 80% under BLV > 6 months) and were included in the analysis, together with 13 hepatologists. The findings revealed key challenges related to patient education, logistical access to medication, and psychological factors affecting motivation. Specifically, 10% had considered discontinuing treatment and 10% admitted to having missed doses. A deeper understanding of these multifactorial determinants may aid in the development of targeted interventions to enhance adherence and achieve personalized therapeutic outcomes for individuals living with HDV.

**Abbreviations**
ALT: alanine aminotransferase
BLV: bulevirtide
CHD: chronic hepatitis delta
NA: nucleos(t)ide analogs

## 1 Introduction

Adherence to antiviral therapy is critical for achieving successful clinical outcomes, particularly in chronic infections such as hepatitis D virus (HDV). Bulevirtide (BLV), the first approved treatment for HDV, has shown robust efficacy in both clinical trials and real‐world settings [1]. However, sustaining long‐term adherence remains challenging due to factors such as the daily subcutaneous administration requirement and the socio‐economic and logistical barriers faced by many patients—especially migrants, who are disproportionately affected by HDV in Europe. Although clinical trials report promising adherence rates alongside improvements in patient‐reported outcomes (PRO) and quality of life during extended treatment [2], and real‐life data confirm BLV's safety and effectiveness in Italy [3], detailed insights into patients' perceptions of treatment are lacking. Understanding behavioural, structural, and psychological barriers to adherence is essential to optimise therapy. The COM‐B model (Capability, Opportunity, Motivation—Behaviour) [4], a validated behavioural framework applied in over 750 studies [5], provides a comprehensive approach to identify such factors and develop targeted interventions [6, 7]. This study applied the COM‐B model to explore adherence challenges among BLV‐treated HDV patients across five hepatology centers in Italy.

## 2 Methods

This was a real‐life, cross‐sectional, multicenter study conducted in Italy. Adult patients with chronic hepatitis D (CHD) receiving BLV were consecutively enrolled between June 1 and September 30, 2024, from five referral centers (Bergamo, Turin, Imola, Naples, and Siracusa). Inclusion criteria included age ≥ 18 years and initiation of BLV therapy before May 31, 2024. Patients completed an anonymous paper survey available in four languages (Italian, English, French, Romanian) during a scheduled clinic visit. Completed surveys were returned anonymously on the same day. The study was conducted in accordance with the Declaration of Helsinki, using only aggregated data and patient consent was implied by completion of an anonymous survey.

The survey captured demographic data (age group, region of residence, country of origin, treatment duration group) and included both structured and open‐ended items aligned with the COM‐B domains:

1. Capability: Patient understanding of treatment procedures and side effect management;
2. Opportunity: Access to healthcare, drug supply and social support;
3. Motivation: Perceptions of treatment benefits, emotional attitudes and commitment.

In parallel, a second survey was administered to hepatologists at the participating centers to collect clinician perspectives on adherence challenges. The 21‐item questionnaire was developed and refined by the authors during a dedicated meeting in Spring 2024. After collecting the anonymous surveys, the same team reconvened to analyze the data, with the support of statisticians, psychologists, and a representative from EpaC, the leading Italian patient advocacy group for liver diseases. Data were analyzed using the COM‐B model to identify key themes and inform potential interventions.

## 3 Results

Of 86 eligible patients, 83 (97%) completed the anonymous patient survey. The cohort had a diverse demographic profile: 35% were over 60 years old, 30% aged 50–60, 22% aged 40–49, and 13% aged 18–39. Nearly half of the participants were Italian (48%), while the remainder originated from Romania (13%), Moldova (11%), Africa (6%), Albania (6%), Ukraine (2%) and Lithuania (2%). The majority resided in Northern (40%), Southern (29%) or Central (19%) Italy. Notably, 12% of participants did not report either their country of origin or their place of residence. Survey responses suggest that both region of residence and country of origin influence patients' perceptions and experiences. At the time of survey completion, almost 80% of patients had been on BLV treatment for over 6 months, 12% for < 3 months and 8% between 3 and 6 months. Thirteen clinicians from the centers completed the parallel physician survey.

### 3.1 Capability

**FIGURE 1:**
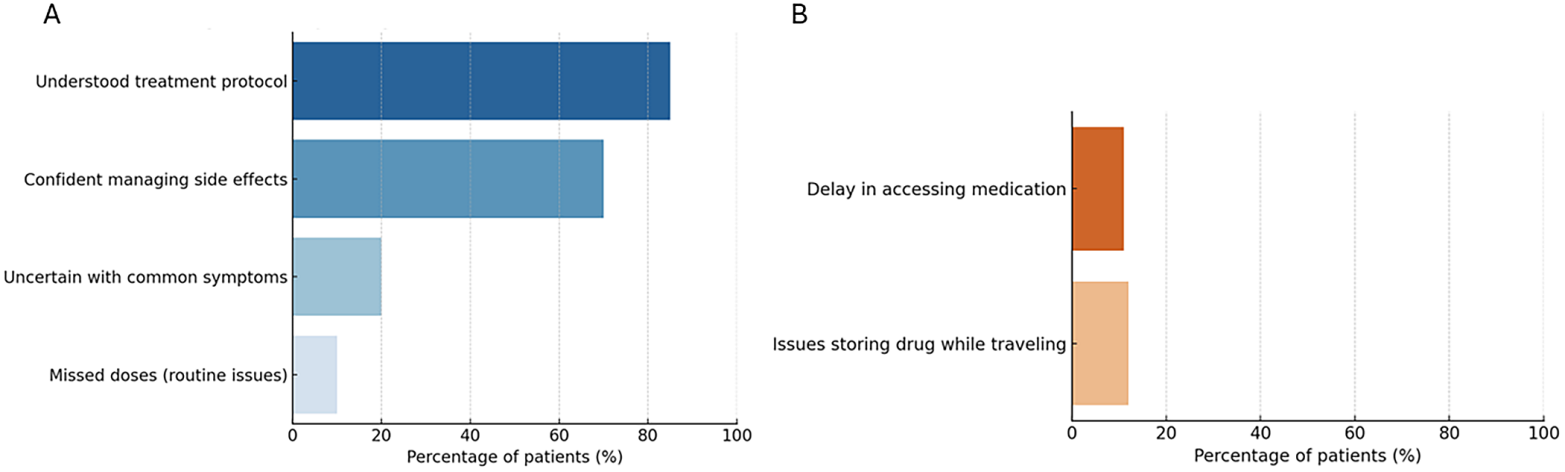
Capability (A) and Opportunity (B)‐related barriers to bulevirtide adherence. Proportion of patients reporting challenges related to treatment understanding, side effect management and daily administration routine.

All patients reported correctly performing BLV administration. However, 10% admitted to skipping doses occasionally, and 13% did not consistently adhere to the scheduled time. Despite 95% stating they had received information about side effects, 20% felt unprepared to manage possible adverse events and 15% lacked a designated healthcare contact (Figure 1). Foreign patients more often report difficulties with treatment adherence and managing side effects. Clinicians reported providing initial training but acknowledged time constraints during early visits limited the depth of instruction. These findings highlight the need for structured education and ongoing reinforcement.

### 3.2 Opportunity

Systemic barriers were identified in drug delivery and diagnostic access. About 11% of patients experienced medication delays, and 12% reported difficulties maintaining cold chain storage during travel. Access to HDV RNA testing and liver function monitoring was inconsistent, particularly in Southern Italy (Figure 1B). Clinicians confirmed regional disparities in drug availability and laboratory services. Some centers had introduced ‘Delta Days’, combining clinical visits, diagnostics and drug dispensing into a single appointment to improve continuity of care, but these initiatives were not widespread.

### 3.3 Motivation

Patients from Africa, Ukraine, Moldova and Romania tend to have less optimistic expectations regarding life expectancy. A subset of patients (12%) reported not perceiving significant benefits from BLV, questioning its value in the absence of symptoms. This group expressed doubts about long‐term outcomes, and some (10%) had considered discontinuing treatment. Notably, motivation often decreased after virological suppression, suggesting a misunderstanding of the treatment goal. Clinicians addressed this by using visual aids (e.g., HDV RNA and ALT trends) to reinforce progress and personalized discussions to maintain engagement.

Moreover, the survey did allow patients to report their own perception of the severity of their liver disease. Although the majority of participants (86%) perceived their liver disease as very or moderately severe, 14% regarded it as only mildly severe or not severe at all; optimism about improvements in daily life was higher among patients who believed their disease was less severe. Of note, the proportion of patients who reported having considered discontinuing treatment was comparable between the two groups.

When comparing the patient and clinician surveys, the main differences emerged in a few key areas: the availability of a contact person in case of therapy‐related questions, which clinicians consider important but are often overlooked by patients; treatment compliance, which patients consistently report as optimal during medical visits but indicate a lack of adherence in 10% of cases in the survey; and treatment goals, which are clear to clinicians but often not fully understood by patients.

## 4 Discussion

Bulevirtide, an entry inhibitor rather than a direct‐acting antiviral, has demonstrated efficacy in registration trials as well as in multiple real‐world studies, mainly involving highly motivated cirrhotic patients with portal hypertension. The majority of patients who initiated BLV monotherapy in recent years in Italy were cirrhotic (100% in the SAVE‐D study [8] and 90% in the ARISTOTELE study [3]). Clinically, the patients included in this survey are the same cohort as those enrolled in the real‐world ARISTOTELE study; therefore, nearly all are cirrhotic, and 100% are receiving concomitant treatment with nucleos(t)ide analogues. BLV therapy presents unique adherence challenges due to its administration mode and the chronic nature of HDV. This is the first study to systematically investigate the determinants of BLV adherence in patients with CHD and revealed several unexpected barriers from both the patient and clinician perspectives. Using the COM‐B model, key domains were identified that influence patient behaviour and adherence.

Capability issues were less about technical skills and more about confidence and education. Patients understood how to administer BLV but were unsure how to respond to side effects or missed doses. Initial education was often brief and lacked follow‐up. Solutions include developing multilingual, patient‐friendly materials (e.g., videos, handouts) and involving caregivers in training sessions.

Opportunity barriers were predominantly structural. Regional inequalities in drug distribution and diagnostic testing limited consistent follow‐up. Patients in the South were disproportionately affected. A centralised, standardised drug delivery and monitoring system could improve equity. Community‐based care models like ‘Delta Days’ could be scaled nationally to enhance support and adherence.

Motivation appeared to wane in asymptomatic patients or those achieving virological suppression. Many viewed the absence of symptoms as a sign that treatment was no longer needed. These patients require ongoing counselling to understand the long‐term goals of therapy. Visual progress tracking, regular feedback and psychological support could help sustain engagement.

Notably, adherence problems were not associated with the technical aspects of injection—100% of patients performed the procedure correctly—but rather with integrating therapy into daily life. Comparisons with other chronic conditions such as diabetes (where insulin adherence is ~42%) underscore that medication burden, even when manageable, can affect long‐term commitment.

This study is the first to systematically apply the COM‐B model to HDV patients receiving BLV in Italy. Its findings align with those of the MYR301 trial, which reported improved PROs during BLV therapy, yet suggest that real‐world adherence may be less robust. The lack of clinical correlation (e.g., disease severity, virological or biochemical response during treatment), as well as the absence of data on education level and employment type, represents limitations of this anonymous survey, along with the relatively small sample size. Nevertheless, the cohort represents approximately one‐sixth of BLV‐treated patients in Italy during the first half of 2024.

An interesting demographic finding was that younger and foreign‐born patients reported more adherence issues, likely due to work instability, cultural differences or recent diagnosis. In contrast, older Italian patients—who often waited years for treatment—were generally more motivated and compliant. This divergence points to the need for culturally sensitive, age‐adapted approaches to patient education and support.

## 5 Conclusion

This study demonstrates the utility of the COM‐B model in identifying behavioural and structural barriers to BLV adherence in HDV patients. Addressing capability gaps through standardised education, reducing opportunity barriers via equitable drug access and diagnostics, and strengthening motivation through personalised engagement strategies are critical steps.

The future of HDV management will depend not only on therapeutic innovations but also on our ability to support patients in sustaining adherence. As new data on optimal treatment duration and virological endpoints emerge, flexible, patient‐centered models of care must be developed. The insights from this study provide a foundation for designing such strategies, ensuring that the clinical benefits of BLV are fully realized across diverse patient populations.

## Author Contributions

**Lorenzo Badia**, **Alessia Ciancio**, **Marco Distefano**, **Antonio Izzi**, **Alessandro Loglio:** conceptualization, data collection and writing. **Alessandro Loglio:** review and editing. **Alessandro Loglio:** supervision. All authors have read and agreed to the published version of the manuscript.

## Conflicts of Interest

Alessandro Loglio: Speaker bureau and received grants from Gilead Sciences and Roche. The other authors declare no conflicts of interest.

## Data Availability

The data that support the findings of this study are available from the corresponding author upon reasonable request.
